# Multiparametric MRI‐based model for prediction of local progression of hepatocellular carcinoma after thermal ablation

**DOI:** 10.1002/cam4.6277

**Published:** 2023-09-11

**Authors:** Chao Chen, Qiuying Han, He Ren, Siyi Wu, Yangyang Li, Jiandong Guo, Xinghai Li, Xiang Liu, Chengzhi Li, Yunfei Tian

**Affiliations:** ^1^ Department of Minimal Invasive Intervention Radiology Ganzhou People's Hospital Ganzhou China; ^2^ Department of Cardiology The First Affiliated Hospital of Jinan university Guanghzhou China; ^3^ Department of Ultrasound The Six Medical Center of Chinese PLA General Hospital Beijing China; ^4^ Department of Interventional Radiology and Vascular Surgery The First Affiliated Hospital of Jinan University Guanghzhou China

**Keywords:** deep learning radiomics, hepatocellular carcinoma, local tumor progression, multiparametric magnetic resonance imaging, risk stratification

## Abstract

**Purpose:**

To develop a deep learning radiomics of multiparametric magnetic resonance imaging (DLRMM)‐based model that incorporates preoperative and postoperative signatures for prediction of local tumor progression (LTP) after thermal ablation (TA) in hepatocellular carcinoma (HCC).

**Methods:**

From May 2017 to October 2021, 417 eligible patients with HCC were retrospectively enrolled from three hospitals (one primary cohort [PC, *n* = 189] and two external test cohorts [ETCs][*n* = 135, 93]). DLRMM features were extracted from T1WI + C, T2WI, and DWI using ResNet18 model. An integrative model incorporating the DLRMM signature with clinicopathologic variables were further built to LTP risk stratification. The performance of these models were compared by areas under receiver operating characteristic curve (AUC) using DeLong test.

**Results:**

A total of 1668 subsequences and 31,536 multiparametric MRI slice including T1WI, T2WI, and DWI were collected simultaneously. The DLRMM signatures were extracted from tumor and ablation zone, respectively. Ablative margin, multiple tumors, and tumor abutting major vessels were regarded as risk factors for LTP in clinical model. The AUC of DLRMM model were 0.864 in PC, 0.843 in ETC1, and 0.858 in ETC2, which was higher significantly than those in clinical model (*p* < 0.001). After integrating clinical variable, DLRMM model obtained significant improvement with AUC of 0.870–0.869 in three cohorts (all, *p* < 0.001), which can provide the risk stratification for overall survival of HCC patients.

**Conclusions:**

The DLRMM model is essential to identify LTP risk of HCC patients who underwent TA and may potentially benefit personalized decision‐making.

## Abstract 2

Findings: In this multicenter study that included 417 patients, a deep learning radiomics model was proposed, which achieved high prediction accuracy with AUC of 0.864‐0.858 across one training and two external testing cohorts, and realized successful patient stratification of high and low local recurrence risk. Meaning: The proposed prediction system may help physicians in therapeutic decision making and surveillance strategy selection for hepatocellular carcinoma patients after thermal ablation in clinical practice.

## 1 INTRODUCTION

Hepatocellular carcinoma (HCC) is the third most leading cause of cancer‐specific mortality globally, and >50% of these cases occurred in China.^1^, ^2^, ^3^ Image‐guided percutaneous thermal ablation (IPTA) are recommended as a first‐line treatment in patients with HCC in early stage (BCLC – A).^4^, ^5^ Moreover, IPTA also was used as a bridge to liver transplantation (LT) during waiting process for the organ source.^6^ Given IPTA has many advantages including less trauma, fewer complication, better repeatability, and cost‐effectiveness, it has become an alternative treatment for HCC patients ineligible for surgery. Previous studies reported that HCC patients underwent IPTA can achieve comparable survival outcomes than surgery.^7^, ^8^ Unfortunately, a approximately 45%–62% of post‐ablation recurrence rate was found in clinical practice.^9^ Especially, early recurrence usually result in poor prognosis, which bring a great challenge for physicians.^10^

Local tumor progression (LTP) is a special type of recurrence after IPTA. Accumulated evidence demonstrates that untreated micrometastases from the primary tumor, the subsequent spread along intrasegmental branches and vascular invasion may contribute to LTP. Although, numerous studies have suggest that LTP is associated with tumor aggressiveness, mainly including >5 cm of tumor diameter, microscopic vascular invasion, poor pathological differentiation, etc., it remains be not consistent owing to tumor heterogeneity and patients' individual differences.^11^, ^12^, ^13^ Among many risk factors, ablative margin (AM) (generally, 5‐mm safe margin beyond tumor margin) plays an important role in LTP. Therefore, a large number of measurement methods of AM have been proposed. For example, Kai Li et al. suggest that 5‐mm AM is recommended for IPTA candidates with single HCC (diameter ≤3 cm), and the AM had a pronounced nonlinear impact on LTP.^14^

Accurate measurement of AM is extremely difficult, which is closely related to image registration before and after ablation. In view of the liver elastic deformation caused by respiratory movement and heat, it is difficult for radiologists to obtain accurate AM. To date, a well‐received biomarker for prediction remains elusive. Recently, the potential power of medical image data is recognized increasingly in the field of oncology.^15^, ^16^ The radiomics and deep learning (DL) as the state‐of‐the‐art quantitation has been used increasing to build models for oncological outcomes prediction.^17^, ^18^, ^19^ Given the high‐throughput extraction of quantitative imaging features derived from multiparametric magnetic resonance imaging (MRI), it helps to improve the prediction ability of LTP after IPTA. Therefore, we attempt to develop and validate an integrative model that incorporates multiparametric MRI signatures and clinical variables for predicting the individual LTP risk, which provide guidance for IPTA indications selection and making therapeutic strategies.

## 2 MATERIALS AND METHODS

This multiinstitutional study protocol was approved by the Ethics Committee of all participating institutions and conducted following the principles of the 1975 Declaration of Helsinki. The requirement for written informed consent was waived because of the retrospective nature of this study.

### 2.1 Study design

**FIGURE 1:**
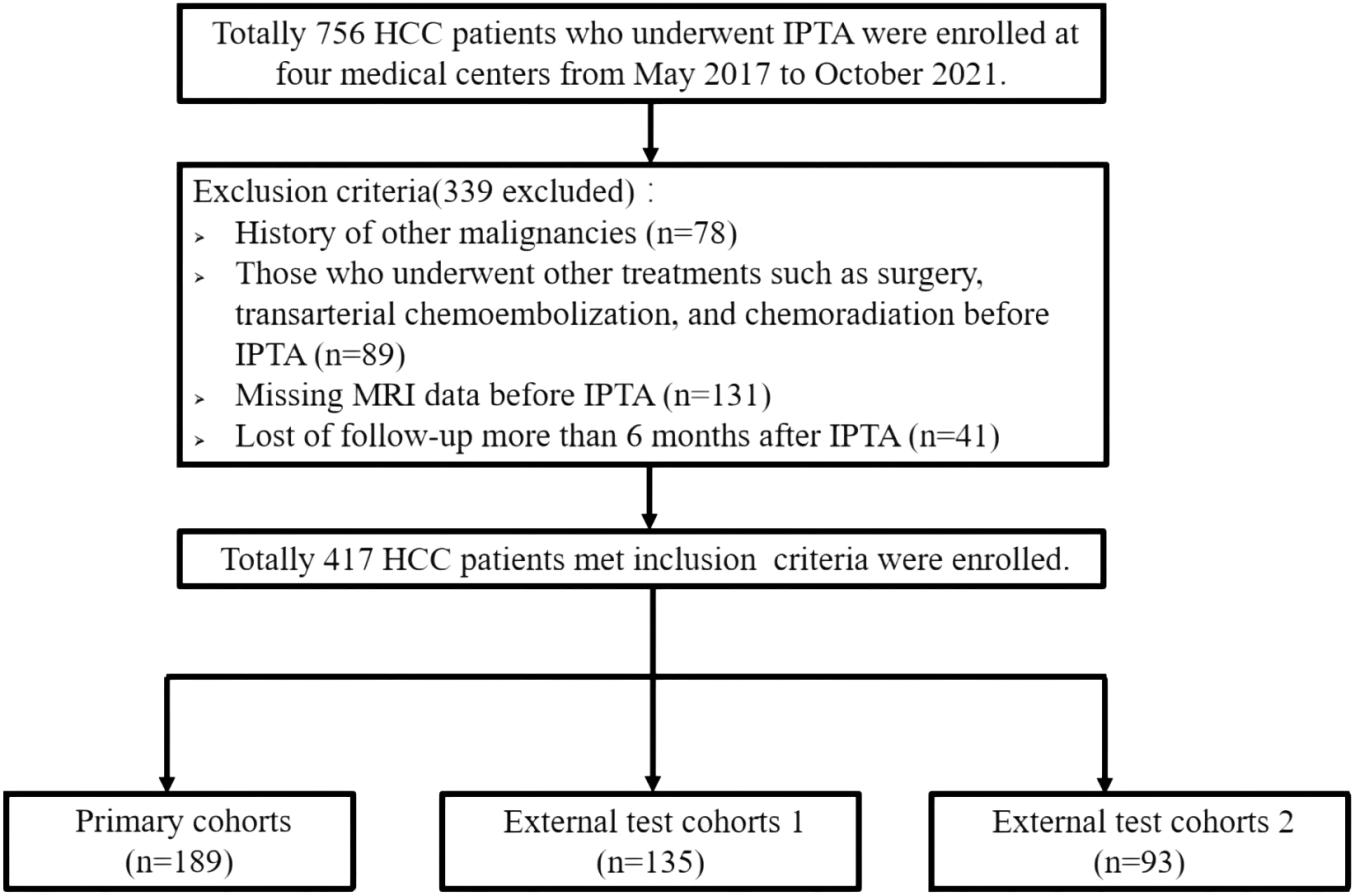
The enrollment pathway of patient with hepatocellular carcinoma who underwent thermal ablation.

From May 2017 to October 2021, a total of 756 patients with early‐stage HCC who subsequently underwent initial IPTA were reviewed. Dynamic‐enhanced MRI were performed before IPTA and the criteria of HCC diagnosed was defined according to the American Association for the Study of Liver Diseases (AASLD). Figure 1. demonstrates the patient enrollment pathway. The inclusion and exclusion criteria are outlined as following: (i) Eastern Cooperative Oncology Group performance (ECOG) scores <2; (ii) Child‐Turcotte‐Pugh grade A or B; (iii) all HCC patients received the enhanced MRI scan 2 weeks before IPTA; (iv) complete ablation was confirmed radiologically. The exclusion criteria were as follows: (i) history of other malignancies; (ii) those who underwent other treatments before IPTA; (iii) missing MRI data before IPTA; (iv) lost of follow‐up more than 6 months after IPTA. The IPTA procedures and equipment was shown in Appendix S1. Finally, a total of 417 patients were enrolled. Among them, 189 was from primary cohort (PC) and the patients in training cohort and validation cohort were assigned according to 4:1 rate in PC. Moreover, 135 were enrolled in external test cohort [ETC] 1 and 93 were enrolled in ETC 2.

### 2.2 Follow‐up and LTP assessment

The follow‐up duration was terminated in October 2022. The serum alpha‐fetoprotein (AFP) and dynamic contrast‐enhanced images were examined again at 1 and 3 months after IPTA and at approximately 3‐ to 6‐month intervals. Local tumor progression (LTP) was defined according to imaging results of abnormal nodular, disseminated, and/or unusual patterns of peripheral enhancement around the ablative site in patients treated with IPTA.^20^ Overall survival (OS) was calculated from the date of initial IPTA to the date of death of any cause or deadline for follow‐up. Sixteen clinical variables were collected according to clinical experience and listed in Appendix S2 and clinical information of missing ratio greater than 20% were excluded. Among these variable, ablative margin (AM) plays a important role in LTP. The AM was defined as the shortest distance from the edge of the tumor to the edge of the ablation zone.

### 2.3 MRI parameters selection

**FIGURE 2:**
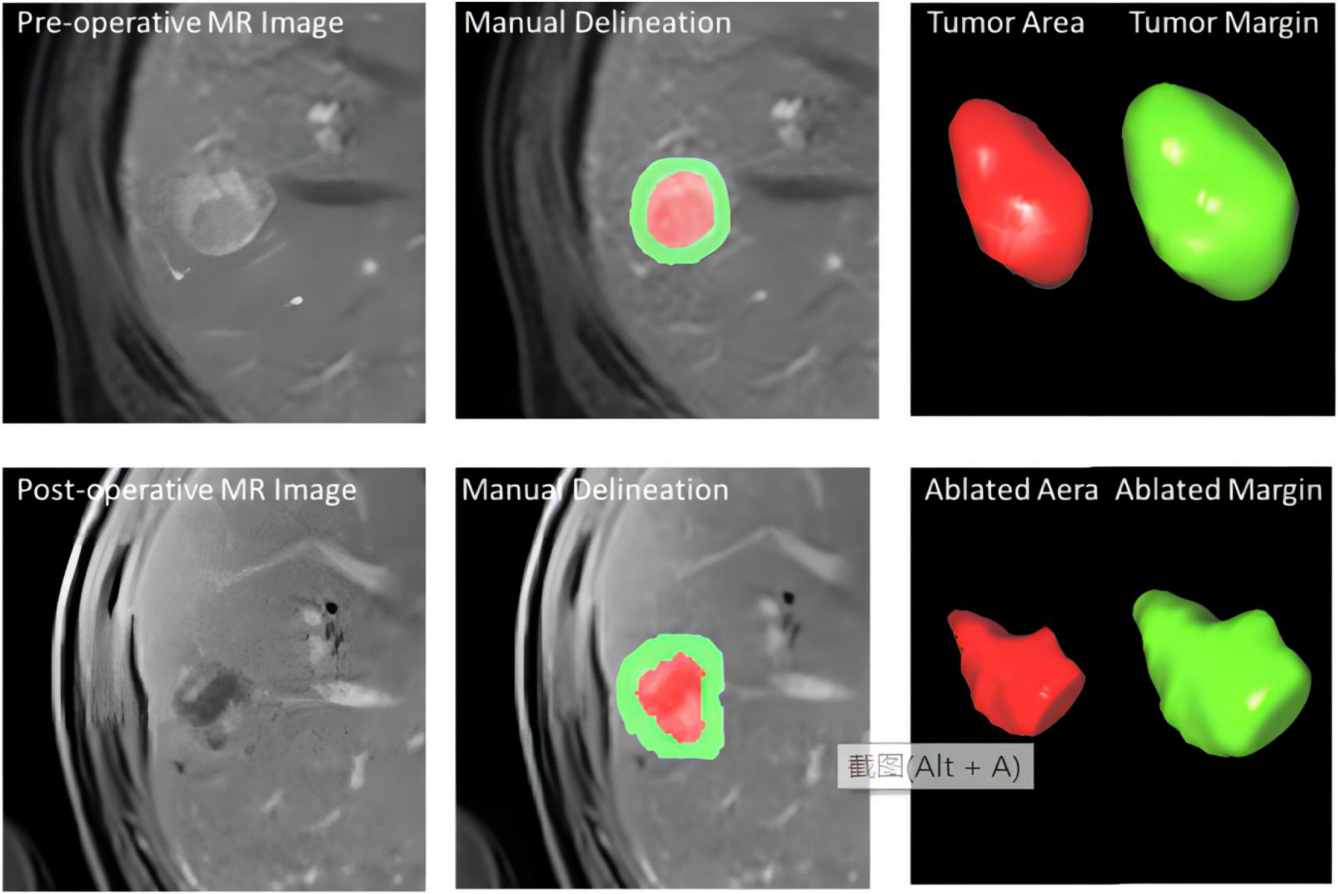
3D reconstruction of delineation of tumor region and ablative region.

The MRI raw data before and after IPTA were taken from the picture archiving and communication system (PACS) database. The multiparametric MRI data (T2WI, T1WI + C, and DWI) of pre‐ablation and T1WI + C of post‐ablation were both collected. MRI scan parameters are described in Table S1. For region of interest (ROI) segmentation, two radiologists (reader 1, C.C., with 10 years of experience of liver imaging experience, and reader 2, J.Z., with 8 years of experience of liver imaging experience) separately delineated the regions including tumor on pre‐ablation multiparametric MRI and ablative region on post‐ablation T1W + C images using an in‐house software coded by Python. On each slice of the T2WI, DWI (b‐value of 800 s/mm^2^), and T1WI + C data. ROIs of HCC nodule were manually drawn along the tumor margin on T2WI (slightly high signal) and T1WI + C (low signal in pre‐enhancement phase, enhancement in arterial phase, washout in the portal vein phase) containing the tumors' chords and burrs, and ROIs were also placed on the high‐signal intensity region on DWI. Extended 5‐mm tumor margin of tumor was automatically reconstructed using erosion and dilation algorithm (MATLAB *imerode* and *imdilate* function). The 2D contour and 3D reconstruction of delineation is shown in Figure. 2.

### 2.4 Feature extraction and selection

Multiparametric MRIs were normalized using Z‐score and nearest interpolation method to the standard normal distribution of image intensity and 1 × 1 × 1 mm^3^ of isotropic spatial resolution. Radiomics features were extracted from above‐mentioned ROI using Pyradiomics package (https://www.radiomics.io/pyradiomics.html). ResNet18 pre‐trained on ImageNet and fine‐tuned on MRIs was used to extract DL features.^21^, ^22^, ^23^, ^24^, ^25^ The univariate analysis was used to compare all features between LTP and non‐LTP patients using Mann–Whitney *U* test. A random forest (RF) based feature selection method named Boruta to screen the most important features to differentiate LTP status.

### 2.5 DL based model construction

To develop and test the deep learning radiomics (DLR) based models, these patients were divided into two cohorts: one primary cohorts (PC) and two external test cohorts (ETCs). Multiparametric MRI features were further explored, and four different MRI signatures including T1WI + C, T2WI, DWI extracted from tumor, and T1WI + C extracted from ablative zone. The generated multichannel feature maps flattened into a one‐dimensional vector were treated as the resulted DLR‐based features (Appendix S2). After all feature extraction and selection (clinical variables, radiomics, and DL signatures), the machine learning methods including Support Vector Machine (SVM), Gradient Boosting Decision Tree (GBDT), Linear Discriminant Analysis (LDA), and Logistic Regression (LR) were used to predict the LTP.^26^ Then, AM based, clinical, DLR and DLR combined with clinical models were built, respectively.

### 2.6 Statistical analysis

Continuous variables were given as mean ± standard deviation (SD) or median with interquartile range (IQR) and compared by the Kruskal–Wallis test. Categorical variables were presented as frequencies with percentages and compared by the chi‐squared test. Univariate and multivariate logistic regression analyses were applied to calculate the hazard ratios (HRs) and corresponding 95% confidence interval (CI). The discrimination, and predictive performance of these models were assessed by areas under receiver operating characteristic curve (AUC) and compared by using DeLong test. The predictive accuracy, sensitivity (SENS), specificity (SPEC), positive predictive value (PPV), and negative predictive value (NPV) were also used to assess the performance of the prediction models. Survival curves were calculated using the Kaplan–Meier method and compared with the log‐rank test.

A two‐tailed *p*‐value of less than 0.05 was considered as statistical significance.

## 3 RESULTS

### 3.1 Patient and tumor characteristics

We trained a DLR model to predict LTP based on pre‐ and post‐ablation multiparametric MRI in PC and performed validation on two independent ETCs. The 417 HCC patients (74 females and 343 males; mean age, 55.4 ± 11.3 years) with 512 lesions (mean diameter, 2.2 ± 0.9 cm) were screened in this study. Among them, 195 patients received radiofrequency ablation (RFA) and 222 patients received microwave ablation (MWA), respectively. The baseline characteristics of the patients between PC and two ETCs are outlined in Table 1. The median follow‐up duration for all patients was 22.5 months (IQR, 11.2–55.3 months). Patients were classified into two categories according to whether LTP occurred. The LTP rate after IPTA was 10.1% (19/189) in PC, 8.8% (12/135) in ETC1 and 14.5% (13/93) in ETC2, respectively. We compared the distribution of different clinical variables between the two groups (LTP vs. non‐LTP). There were statistically significant association between ER and clinical variables including gender, AM, tumor number and adjacent to the major vessels in PC (all, *p* < 0.05).

**TABLE 1:** Baseline characteristics of patients with HCC who received IPTA in three cohorts.

Variables	Primary cohort (*n* = 189)		*p* Value	External test cohort 1 (*n* = 135)		*p* Value	External test cohort 1 (*n* = 93)		*p* Value
	LTP (*n* = 19)	Non‐LTP (*n* = 170)		LTP (*n* = 12)	Non‐LTP (*n* = 123)		LTP (*n* = 13)	Non‐LTP (*n* = 80)	
Demographics									
Age (y)	55.9 ± 11.1	55.8 ± 11.0	0.915	54.5 ± 10.6	55.6 ± 9.9	0.665	54.5 ± 11.2	55.9 ± 11.6	0.448
Gender			**0.013** *			1.000			0.813
Female	2 (10.4)	42 (24.6)		2 (16.6)	16 (16.3)		2 (15.4)	15 (18.6)	
Male	17 (86.9)	128 (75.4)		10 (83.4)	107 (83.7)		11 (84.6)	65 (81.4)	
BMI	21.2 ± 5.6	21.4 ± 4.9	0.562	21.5 ± 7.2	22.1 ± 3.3	0.448	22.8 ± 7.5	21.7 ± 3.7	0.289
PS			1.000			1.000			1.000
ECOG 0	18 (94.7)	161 (94.7)		12 (100)	122 (99.2)		12 (92.3)	74 (92.5)	
ECOG 1	1 (5.3)	9 (5.4)		0 (0)	1 (0.8)		1 (7.7)	6 (7.5)	
Comorbidities			0.399			0.817			0.141
Absence	14 (72.7)	114 (67.0)		10 (83.4)	105 (86.2)		8 (61.5)	62 (77.5)	
Presence	5 (27.3)	56 (33.0)		2 (16.6)	18 (14.6)		5 (38.5)	18 (22.5)	
HBV			0.771			**0.023** *			0.495
Absence	30 (39.0)	46 (41.1)		3 (25)	11 (8.9)		2 (15.4)	9 (11.4)	
Presence	47 (61.0)	66 (58.9)		9 (75)	112 (91.1)		11 (84.6)	71 (88.6)	
CTP grade			0.862			0.683			0.724
A	18 (94.7)	154 (89.3)		11 (91.7)	108 (87.8)		12 (92.3)	71 (88.7)	
B	1 (5.3)	16 (9.8)		1 (9.3)	15 (12.2)		1 (7.7)	9 (11.3)	
Tumor size (cm)	2.3 ± 1.0	2.4 ± 1.0	0.452	2.1 ± 0.5	1.9 ± 0.6	0.464	2.0 ± 0.9	2.1 ± 1.1	0.912
No. of tumor			**0.003** *			1.000			**<0.001** *
Single	16 (84.2)	165 (97.1)		12 (100)	123 (100)		8 (61.5)	74 (92.5)	
Multiple	3 (15.8)	5 (2.9)		0 (0)	0 (0)		5 (38.5)	6 (7.5)	
Safe margin			**0.009** *			0.576			**0.024** *
>5 mm	16 (84.2)	128 (75)		9 (75)	92 (74.8)		9 (69.2)	71 (88.7)	
≤5 mm	3 (15.8)	42 (25)		3 (25)	31 (25.2)		4 (30.8)	9 (11.3)	
Adjacent to the major vessels			**0.016** *			**<0.001** *			**0.042** *
Absence	12 (63.6)	135 (79.5)		7 (58.3)	121 (98.3)		6 (46.2)	53 (66.3)	
Presence	7 (36.4)	35 (20.5)		5 (41.7)	2 (1.7)		7 (53.8)	27 (33.7)	
AFP (ng/ml)	720.6	253.6	0.174	154.3	242.3	0.502	458.8	197.8	0.342
ALB	41.9 ± 4.1	42.1 ± 4.7	0.776	40.4 ± 4.4	40.0 ± 4.9	0.625	39.0 ± 5.1	40.3 ± 5.5	0.571
AST (U/L)	42.4	30.9	0.452	–	–	–	82.5	86.3	0.771
ALT (U/L)	48.1	33.7	0.104	–	–	–	90.4	65.2	**0.015** *
TBIL (μmol/L)	15.3	15.9	0.623	20.8	16.4	0.091	17.3	20.5	0.238

*Note*: Data are number of patients; data in parentheses are percentage unless otherwise indicated. Data in bracket were percent of patients. The data in two groups were compared by using the chi‐squared test. Non‐normally distributed data are represented by median.

Abbreviations: AFP, α‐fetoprotein; ALB, albumin; ALT, alanine aminotransferase; AST, aspartate aminotransferase; BMI, body mass index; ECOG, Eastern Cooperative Oncology Group; HBV, viral hepatitis type B; IPTA; Image‐guided percutaneous thermal ablation; LTP, local tumor progression; PS, performance status; TBIL, total bilirubin.

* *p* value <0.05.

Bold indicates statistically significant.

### 3.2 Feature extraction and selection

**FIGURE 3:**
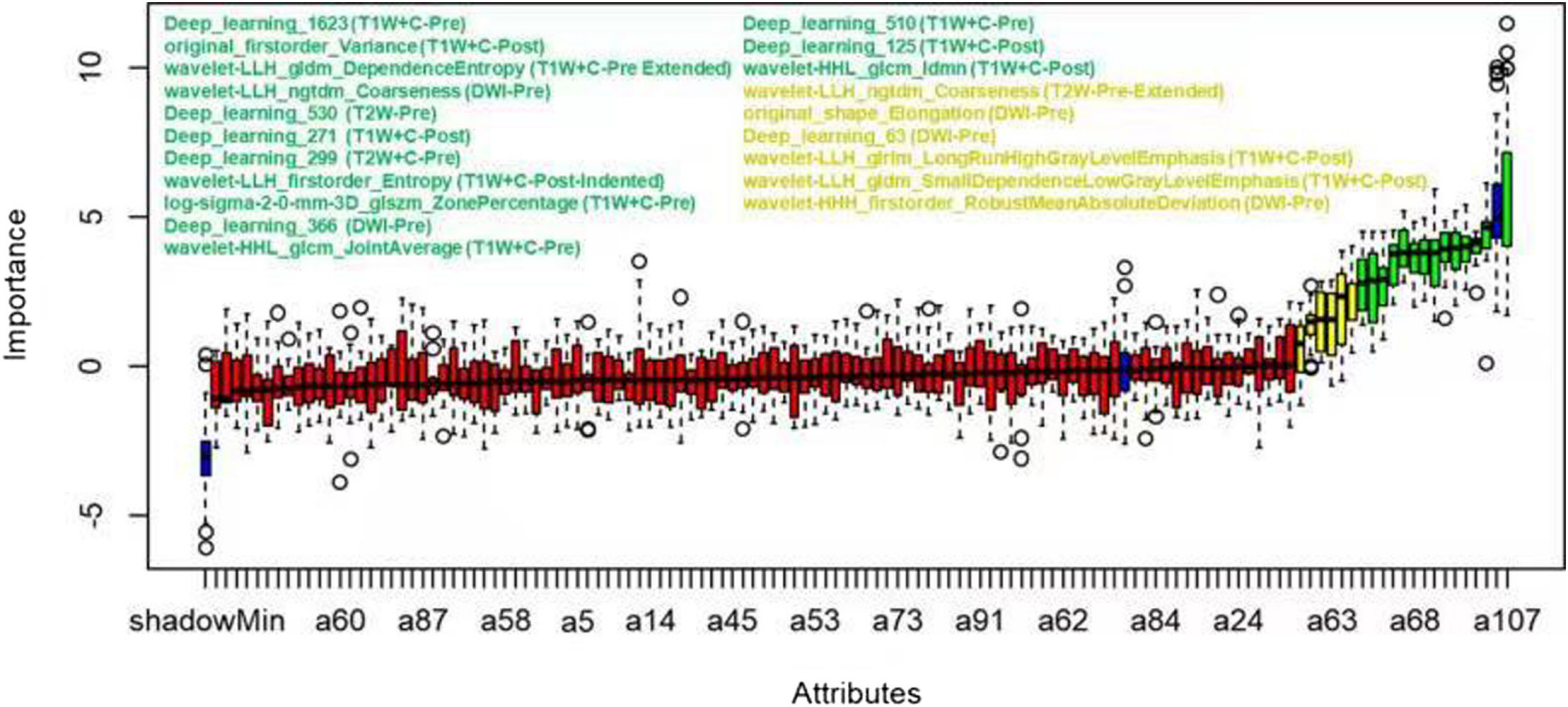
The feature extraction and selection from multiparametric MRI based on deep learning radiomics model.

The statistically significant (*p* < 0.10) indicators of LTP in the univariable regression analysis were input in the multivariable regression model by backward stepwise elimination. Multivariate regression analysis found No. of tumor (OR: 2.263; 95% CI: 1.536–3.334; *p* < 0.001), AM (OR: 1.632; 95% CI: 1.099–2.425; *p* = 0.015), and location abutting major vessels (OR: 10.251; 95% CI: 6.148–17.091; *p* < 0.001) were significantly associated with LTP in the PC (Table 2). Moreover, 15,561 radiomics and 8192 DL features were extracted for each patient. After the reliability evaluation and univariate analysis, 175 features with were selected. Then, Boruta method was performed to screen out 20 features for DLR model construction. The feature extraction and selection in details are shown in Figure 3. Notably, the radiomic feature of T1W + C had the highest ratio in all feature groups (5/11, 45.4% for pre‐DLR; 9/14, 64.3% for post‐DLR; 13/20, 65% for DLR), indicating the MR modality of T1WI + C may contain more abundant information on the prediction of LTP status. Moreover, the *original_firstorder_Variance* was always the top three most powerful predictive features in each group, which may be due to it reflects the heterogeneity of tumors.

**TABLE 2:** Prognostic factors with univariate and multivariate regression for LTP.

Variables	Univariate		Multivariate	
	HR (95% CI)	*p* value	HR (95% CI)	*p* value
Age (years)	1.004 (0.989–0.102)	0.596		
Sex (Female vs. Male)	1.389 (1.170–1.770)	0.013*	1.236 (0.845–1.521)	0.102
Child‐Pugh class (A vs. B)	1.681 (0.692–4.085)	0.251		
Liver disease (HCC vs. Other)	0.748 (0.523–1.070)	0.112		
Cirrhosis (Present vs. Absent)	1.477 (0.963–2.264)	0.074		
Serum bilirubin (μmol/L)	0.990 (0.967–1.013)	0.384		
AFP (ng/ml)	1.000 (0.999–1.001)	0.310		
Multiple tumor (Present vs. Absent)	2.065 (1.391–3.065)	<0.001*	2.263 (1.536–3.334)	<0.001*
Safe margin (Present vs. Absent)	1.568 (1.050–2.341)	0.028*	1.632 (1.099–2.425)	0.015*
Tumor abutting major vessels (Present vs. Absent)	10.960 (6.497–18.489)	<0.001*	10.251 (6.148–17.091)	<0.001*

Abbreviations: AFP, alpha fetoprotein; HBV, hepatitis B virus; LTP, Local tumor progression.

* Statistically significant.

### 3.3 Different predictive models construction

As a result, SVM method can achieve the highest AUC value (0.793 for T2WI, 0.801 for T1WI + C, 0.790 for DWI, and 0.814 for DLR, respectively, in PC) for classifying LTP on all MR sequences than that in other ML classifiers (Table S2). Thus, the SVM was chosen as the classifier in the subsequent study. AM were classified into two categories (≤5 mm and >5 mm). No. of tumor, AM, and location abutting major vessels were used to build the clinical model for prediction of LTP. DLR model based on multiparametric MRI features extracted from tumor and ablative areas was bulid and these significant predictors were added to DLR models for development of DLR‐clinical model, described by the formula: *Y* = −10.523 + 3.345 × location abutting major vessels (0: Absent; 1: Present) + 4.125 × AM (0: ≤5 mm; 1: >5 mm) +4.982 × No. of tumor (0:Single; 1:Multiple) + 7.850 × DLR score.

### 3.4 Performance evaluation of all models

The AUC, ACC, accuracy, SENS, SPEC, PPV, and NPV of all predictive models were shown in Table 3. The AUC of the AM for predicting LTP was 0.781 (95% CI: 0.716–0.846) in the PC, 0.787 (95% CI: 0.722–0.851) in the ETC1, and 0.773 (95% CI: 0.701–0.846) in the ETC2, demonstrating a well‐receiving predictive ability. Clinical model including AM has a better AUC than that of AM alone, and the AUC was 0.801 (95% CI: 0.750–0.852) in the PC, 0.799 (95% CI: 0.763–0.835) in the ETC1, and 0.804 (95% CI: 0.719–0.889) in the ETC2, respectively. The AUC of DLR model was 0.864 (95% CI: 0.812–0.928) in the PC, 0.843 (95% CI: 0.809–0.891) in the ETC1, and 0.858 (95% CI: 0.811–0.904) in the ETC2, respectively. The DLR‐clinical model can improve significantly the predictive ability with AUC of 0.871 (95% CI, 0.823–0.928) in PC, 0.869 (95% CI, 0.833–0.895) in ETC 1, and 0.869 (95% CI, 0.816–0.910) in ETC 2, respectively.

**TABLE 3:** The performance comparison of different models.

Models	Cohorts	AUC (95% CI)	ACC	SENS	SPEC	PPV	NPV
Ablative margin	PC	0.781 (0.716–0.846)	0.741	0.844	0.670	0.637	0.862
	ETC 1	0.787 (0.722–0.851)	0.741	0.579	0.767	0.289	0.918
	ETC 2	0.773 (0.701–0.846)	0.785	0.755	0.818	0.822	0.750
Clinical	PC	0.801 (0.750–0.852)	0.815	0.896	0.759	0.719	0.914
	ETC 1	0.799 (0.763–0.835)	0.807	0.474	0.862	0.360	0.909
	ETC 2	0.804 (0.719–0.889)	0.796	0.776	0.818	0.826	0.766
DLR	PC	0.864 (0.826–0.902)	0.873	0.792	0.929	0.884	0.867
	ETC 1	0.843 (0.795–0.891)	0.859	0.947	0.845	0.500	0.990
	ETC 2	0.858 (0.805–0.910)	0.860	0.816	0.909	0.909	0.816
DLR‐Clinical	PC	0.871 (0.823–0.928)	0.889	0.818	0.938	0.900	0.882
	ETC 1	0.869 (0.833–0.895)	0.874	0.947	0.862	0.529	0.990
	ETC 2	0.869 (0.816–0.910)	0.871	0.816	0.932	0.930	0.820

*Note*: Numbers in parentheses are the 95% confidence interval.

Abbreviations: ACC, accuracy; AUC, areas under receiver operating characteristic curve; DLR, deep learning radiomics; ETC, external test cohort; NPV, negative predictive value; PC, primary cohort; PPV, positive predictive value; SENS, sensitivity; SPEC, specificity.

### 3.5 Risk stratification based on DLR‐clinical model

**FIGURE 4:**
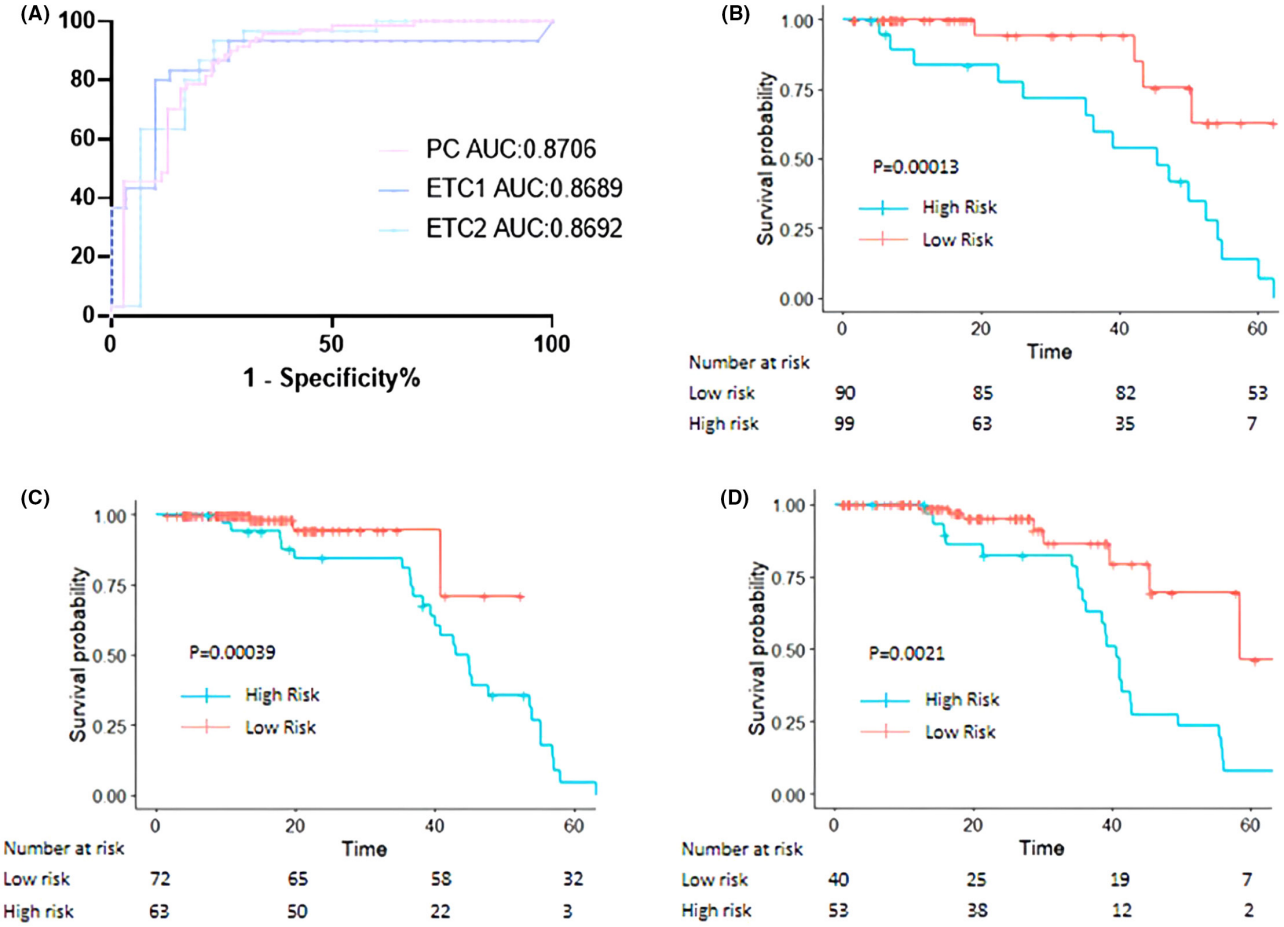
Overall survival (OS) of HCC patients is stratified based on deep learning radiomics combined with clinical model. (A) the AUC comparison between three cohorts; (B) OS comparison between low‐risk group and high‐risk group in the primary cohort; (C) OS comparison between low‐risk group and high‐risk group in the external test cohort 1; (D) OS comparison between low‐risk group and high‐risk group in the external test cohort 2.

The AUC of DLR‐clinical model in PC, ETC1 and ETC2 were compared and shown in Figure 4A. The calibration curves of the DLR‐clinical model in PC, ETC1, and ETC2, which demonstrated good agreement between the predicted LTP probabilities computed by the DLR‐clinical model and the actual LTP probabilities after IPTA. The HCC patients who underwent IPTA were stratified into low‐ and high‐risk subgroups according to the hazard of LTP obtained by risk scores. Using X‐tile software, the optimum stratification threshold was 0.89. The ratios of high‐risk patients in PC, ETC1, and ETC2 were 47.6%, 46.7%, and 57.0%, respectively. Then, Kaplan–Meier survival analysis was performed and it showed significant difference for OS between low‐ and high‐risk in all cohorts (Figure 4B–D). The cumulative 1‐, 3‐, and 5‐ years OS rates were 100%, 94.5%, and 53.4%, in low‐risk group, which were significantly higher than 93.6%, 52.6%, and 11.0% in high‐risk group in PC (*p* < 0.001). The cumulative 1‐, 3‐, and 5‐ years OS rates were 100%, 84.5%, and 69.4%, in low‐risk group, which were significantly higher than 97.6%, 59.6%, and 8.0% in high‐risk group in ETC1 (*p* < 0.001). The cumulative 1‐, 3‐, 5‐ years OS rates were 100%, 87.5%, and 49.4%, in low‐risk group, which were significantly higher than 100%, 55.4%, and 12.4% in high‐risk group in ETC 2 (*p* = 0.002).

## 4 DISCUSSION

Over the last decade, with the development of ablation systems with water cooling cycles and utilizing artificial ascites, larger ablation zone and less heat‐sink effect for these IPTA technique can be found commonly, furthermore, the satisfactory treatment results and overall survival are easier to obtain.^7^, ^8^, ^11^ Nonetheless, LTP after IPTA remains hinder severely survival benefit mainly due to tumor heterogeneity. In this study, 10.6% HCC patients occurred LTP after IPTA with dismal survival. Therefore, the accurate individualized LTP risk estimates play an important role in assisting surveillance regimen and treatment decisions when patients received IPTA treatment. To solve this issue, we built and validated the DLR model for predicting LTP, as well as a integrative model comprising the DLR signature and clinical variables for LTP risk stratification, all of which show very high performance.

The finding of this study mainly included the following four aspects: (i) to the best of our knowledge, this is the first study that the ablation‐related multiparametric MRI data of a consecutive patients with early HCC were collected simultaneously at multiple high‐volume institutions, and used to develop and validate the DLR models based on for predicting LTP after IPTA; (ii) the predictive performance of DLR model based on multiparametric MRI combining feature between tumor and ablation area that select the more image information by supervised DL according to their reproducibility, which outperformed significantly clinical model; (iii) the integrative model comprising the DLR signatures and clinical variables were built and externally validated, that yields the optimal predictive performance among all models; (iv) this study provides evidence for predicting LTP in patients with HCC underwent IPTA, and it is potentially an important major step toward the availability of robust integrative model for the risk assessment and management in HCC patients.

In this study, four different classification methods including SVM, GBDT, LDA, and LR were tested, respectively. SVM showed superior performance among them and we adopt it for the following experiments. Previous studies indicated the radiomic features are enable to quantify tumor heterogeneity by extracting subtle imaging intensity variations, voxel arrangements, and using transformation such as wavelet to represent features at multiple scales.^15^ Moreover, DL was widely used in natural and medical image analysis^16^ and data‐driven way of learning features can be an addition to the radiomic due to its manually designed feature extraction approach. Therefore, we not only used radiomics features, but also integrated DL features to build this predictive model for LTP. The potential value of multiparameter, and multidimensional MRI data have been increasingly received and applied in the precision medicine era. Liu ZY et al. proposed a RMM model for pretreatment prediction of pathologic complete response (pCR) to neoadjuvant chemotherapy (NAC) in breast cancer, yielded an AUC value of 0.86, which was significantly higher than that of clinical model.^27^ Similarly, our proposed DLR models (AUC, 0.864 for PC; 0.843 for ETC1 and 0.858 for ETC2) significantly outperformed the clinical model (*p* < 0.001). Then, the integrative model comprising DLR signatures and clinical variables including number of tumor, safe margin, and location abutting major vessels improved the performance of DLR model, with AUC of 0.871 in PC, 0.869 in ETC1, and 0.869 in ETC2.

Previous reports have been conducted to analyze the risk factors and patterns of recurrence after IPTA.^11^, ^12^, ^13^ Among them, location abutting major vessel is regarded as an important risk indicator, whereas, less than 5‐mm of ablation margin was also associated with LTP. Their results implicated that the high temperature of IPTA not only inactivates carcinoma cells (the temperature at the edge of the ablation zone is lower than that at the center), but may also increase intratumor pressure and intrahepatic metastasize probability through major blood vessels. In addition, a plethora of studies have clarified that tumor microenvironment (TME) play an important role in the initiation and progression of carcinoma during the process of the invasion‐metastasis cascade.^28^, ^29^, ^30^, ^31^ Some normal tissues subject to chronic inflammation easily caused a high cancer incidence. Therefore, any therapeutic methods lies in the tumor stroma all may impart significant influences on tumor progression. As a treatment modality for inactivating in situ tumor, IPTA treatment mainly relies on the regulation of cold or hot temperatures in the tumor area to cause degeneration and necrosis of tumor cells. Correspondingly, TME has also undergone certain changes followed by this operation. Notably, the tumor margin as a crucial gathering place in the TME are highly active and interactive with the tumor itself. There are two key reasons for explaining the importance of tumor margin as following; first, immature myeloid cells often gather here to prevent differentiation of antigen‐presenting DCs, which result in tumor immune evasion. Moreover, CAFs are similarly abundant at the tumor margin where they release pro‐invasive factors for tumor cells and participate in a TGF‐β/PDGF signaling crosstalk with tumor cells. Second, low oxygen environment is also a major driving force for stromal cell behavior and tumor progression. Given oxygen is largely available at the periphery of tumor rather than center, the thermal ablation mechanism will quickly cause the lack of oxygen around the tumor area and close the tiny blood‐supplying arteries around the tumor to further reduce oxygen delivery. To confirm the above hypothesis, we added multiparametric MRI signatures of tumor margin and ablation area. Results also demonstrated that adding these features can boost the predictive performance of DLR models and the discrimination of DLR models present improvement to a certain degree, also verifing the hypothesis of previous studies.

To improve the multidisciplinary treatment of HCC and reduce LTP rate, the HCC patients at high risk of therapeutic strategies including preventive and adjuvant therapy (e.g., LT, preventive transarterial chemoembolization [TACE], multitargeted tyrosine kinase inhibitors [TKIs], and immune therapy).^32^, ^33^ LT is the most effective treatment to prevent recurrence, which allows the optimal allocation of scarce organs and provides comparable survival outcomes to those of upfront LT. TACE after IPTA have the characteristics of minimally invasive and repetitive operations, further minimizing the blood supply of the intrahepatic HCC, and it effectively prevent the LTP of HCC. Our DLR model could affect both the use of adjuvant treatment and the preventive strategy by individualizing management according to the two risk (high/low) profiles for LTP before IPTA. By using the DLR‐based model, we advocate upfront TACE for high‐risk patients; utilizing this model allows prophylactic and pre‐emptive enlistment of high‐risk patients for TACE, TKIs, and PD‐1 before the LTP is identified. In addition, the DLR‐based integrative model may facilitate individualized surveillance policy.

There are certain limitations to our study. First, the sample size of this study was relatively small, the DLR integrative model may be mildly overfitted. Therefore, we must continue to collect MRI data to further confirm the predictive ability and robustness of the DLR based integrative model; Second, the parameters of MRI machine, IPTA technology selection (MWA or RFA) were different from multiple hospitals, these factors may influence the predictive performance of these DLR models; Third, we did not collect other potential molecular biomarkers, including NLR, MMP7, MUC1, and genomic information. The accuracy and robustness of the DLR‐based integrative model may be improved if combined with the above‐mentioned factors.

In conclusion, the easy‐to‐use integrative model consisting of MRI signatures and clinical variables exhibited adequate performance, which was capable of individualized predictive ability that could stratify HCC patients underwent IPTA into different LTP risk groups. Therefore, this DLR‐based tool may help physicians in decision making after IPTA for early HCC in clinical practice and trials. In addition, external tests and designing prospective clinical trials should be performed to obtain more solid and reliable evidence in subsequent studies.

## AUTHOR CONTRIBUTIONS

**Chao Chen:** Writing – original draft (equal). **Qiuying Han:** Writing – original draft (equal). **He Ren:** Data curation (equal). **Siyi Wu:** Formal analysis (equal). **Yangyang Li:** Data curation (equal). **Jiandong Guo:** Validation (equal). **Xinghai Li:** Investigation (equal). **Xiang Liu:** Supervision (equal). **Chengzhi Li:** Writing – review and editing (equal). **Yunfei Tian:** Writing – review and editing (equal).

## FUNDING INFORMATION

None.

## CONFLICT OF INTEREST STATEMENT

The authors of this manuscript declare no relationships with any companies, whose products or services may be related to the subject matter of the article.

## Supporting information


Appendix S1

## Data Availability

The data that support the findings of this study are available from the corresponding author upon reasonable request.
