# Supplementary material for: Multiparametric MRI‐based model for prediction of local progression of hepatocellular carcinoma after thermal ablation
Source: Cancer Med. 2023 Sep 11;12(17):17529–40. doi: 10.1002/cam4.6277 (PMC10524055; doi:10.1002/cam4.6277)
Supplement: Supplementary file 1 — Appendix S1 [file CAM4-12-17529-s001.docx]

**Multiparametric MRI Based Model for Prediction Local Pprogression of Hepatocellular Carcinoma After Thermal Ablation**

**Supplementary Materials**

1. **Supplementary Methods**
   1. Equipment and procedure of IPTA.
   2. Clinical data selection.
   3. Deep learning based model building.
2. **Supplementary Tables**
   1. **Table S1**. MRI protocols used in this study.
   2. **Table S2**. Performance for different classifiers on pre-ablation MR sequences

in primary cohorts.

**1.Supplementary Methods**

**Appendix S1. Equipment and Procedure of IPTA**

The microwave equipment unit (KY-2000; Kangyou Medical, Nanjing, China) was capable of producing 100 W of power at 2,450 MHz. The cool tip needle antenna had a diameter of 1.9 mm (15 gauge) and a length of 18 cm. The radiofrequency ablation equipment was commercially available electrode system with generators (Cool-tip RF System [Covidien, Colorado, MA, USA]) and an internally cooled, 17-gauge, 15 cm, electrode, with a 2 or 3 cm long exposed metallic tip. Before treatment, all patients were scanned using contrast-enhanced computed tomography (CT), magnetic resonance imaging (MRI) and ultrasound (US), and an appropriate puncture route was chosen by ultrasound. With the patient under moderate sedation and local anesthesia, Image-guided percutaneous thermal ablation (IPTA) was performed by three interventional radiologists. After application of local anesthesia with 1% lidocaine (Yi you, Beijing, China), US-guided biopsy was performed in 2–3 separate punctures using an automatic biopsy gun with an 18-gauge cutting needle. Subsequently, the microwave antenna was percutaneously inserted into the tumor and placed on the desired location under US guidance. A power output of 50 W for 10 minutes was routinely used during MWA. After all the punctures, intravenous anesthesia with a combination of propofol (Diprivan; Zeneca Pharmaceuticals, Wilmington, Del) and ketamine (Shuang he Pharmaceuticals, Beijing, China) was administered via the peripheral vein. If the heat-generated hyperechoic water vapor did not completely encompass the entire tumor, prolonged microwave emission was applied until the desired temperature was reached. If the tumor was adjacent to bowel, gallbladder or other important tissues, a 21gauge thermocouple was inserted close to these tissues for real-time temperature monitoring during ablation. One session was permitted in single smaller tumor (diameter < 2cm). And several sessions performed repeatedly until complete margin was confirmed with CT or MRI.

**Appendix S2. Clinical Data Selection**

Sixteen clinical variables were collected according to clinical experience as following (1) demographic variables (age, gender, BMI, comorbidities (i.e., hypertension, diabetes, heart disease, etc), etiology, cirrhosis and Child-Turcotte-Pugh (CTP) grade; (2) image feature (maximal tumor diameter, number, ablative margin[1] and location abutting major vessels, which was defined as a nodule located less than 5 mm from either the major hepatic veins, portal vein or inferior vena cava); (3) laboratory findings (alpha-fetoprotein [AFP], serum albumin [ALB], serum total bilirubin [STB], aspartate aminotransferase [AST] and alanine aminotransferase [ALT]).

**Appendix S3. Deep Learning based Model Building**

In this study, we used the pre-trained neural network ResNet18 to extract abundant image features, which was regarded as an addition to the radiomic features. To increase the ease of the use for the proposed DLR model, we took the pre-trained ResNet18 model as a feature extractor not fine-tuned it on images from the primary cohort. Consider that the last two layers of ResNet18 were average pooling layer and fully connected layer which yielded into a classification task, we took feature maps before these two layers[2-4]. Then, the feature map was flattened into 1D vectors to make the deep learning features. The input of ResNet18 was image bounding boxes with size of 64×64. The input images came from tumor region on pre-T1W+C, pre-T2W and pre-DWI MR sequences and ablation region on post-T1W+C MR sequence. Note that the input of ResNet18 had three channels, we copied the channel of each gray-level image to form the three-channel image. The output of ResNet18 was 512-channel feature map with size of 2×2, thus resulted in 2048 (2×2×512) deep learning features for each image bounding box.

The network structure of ResNet18 without last two layers, which was constructed of 2D convolutional layer (2D conv), rectified linear unit layer (ReLU), batch normalization layer (BN), max-pooling layer, global average pooling layer and fully connected layer (FC)[5].

**Network Training**

The loss function of the network is shown below:

$$L\left( w \right)=\frac{1}{N}\sum_{n=1}^{N} \left[ y_{n}\log p_{n}+\left( 1-y_{n} \right)\log\left( 1-y_{n} \right) \right]$$

where $p_{n}$ is the predicted probability of ER status and $y_{n}$ is the ground truth label that 0 represents non-ER and 1 represents ER.

We used Adam [6] as the optimizer with a batch size of 32. We used the learning rate of 0.0001 and trained for 200 epochs. Data augmentation including random rotation, flip, and crop was applied to reduce the risk of overfitting. We then adopted 10-fold cross validation find best hyper-parameters setting.

**References**

1 An C, Jiang YQ, Huang ZM, Gu YK, Zhang TQ, Ma L*, et al.* Assessment of Ablative Margin After Microwave Ablation for Hepatocellular Carcinoma Using Deep Learning-Based Deformable Image Registration. Front Oncol 2020;**10**.

2 Olivier R, Cao H. Nearest neighbor value interpolation. Int J Adv Comput Sci Appl (USA) 2012;**3**:25-30.

3 van Griethuysen JJM, Fedorov A, Parmar C, Hosny A, Aucoin N, Narayan V*, et al.* Computational Radiomics System to Decode the Radiographic Phenotype. Cancer Res 2017;**77**:E104-E7.

4 He KM, Zhang XY, Ren SQ, Sun J. Deep Residual Learning for Image Recognition. Proc Cvpr Ieee 2016:770-8.

5 Deng J, Dong W, Socher R, Li L, Kai L, Li F-F. ImageNet: A large-scale hierarchical image database. 2009 IEEE Conference on Computer Vision and Pattern Recognition, 2009:248-55.

6 Kingma D, Science JBJC. Adam: A Method for Stochastic Optimization. 2014.

**Table S1**. MRI protocols and parameters

| **Scanner** | **Patients No.** | **Sequence** | **TR/TE**  **(ms)** | **FOV**  **(mm)** | **Matrix** | **Slice Thickness (mm)** | **Slice Gap**  **(mm)** | **Slices** | **Flip Angle** | **Acquisition**  **Time (min)** | **Scans** |
| --- | --- | --- | --- | --- | --- | --- | --- | --- | --- | --- | --- |
| GE 3.0T  (Pioneer) | 121 | T1W+C | 4.7/1.7 | 320×320 | 384×384 | 2 | 2 | 50 | 9° | 4min25s | 6 |
|  |  | T2W | 4000/83.2 | 340×320 | 364×364 | 6 | 1 | 30 | 90° | 2min20s | 1 |
|  |  | DWI | 5244/66 | 320×320 | 240×240 | 6 | 1 | 24 | 90° | 1min35s | 1 |
| Siemens 3.0T  (Trio) | 68 | T1W+C | 5.2/1.6 | 320×320 | 384×384 | 1.5 | 2 | 72 | 12° | 5min3s | 6 |
|  |  | T2W | 3202/67 | 320×320 | 320×320 | 5 | 1 | 30 | 90° | 2min10s | 1 |
|  |  | DWI | 6000/55 | 340×340 | 200×200 | 5 | 1 | 30 | 90° | 1min20s | 1 |
| United imaging 3.0T  (UMR 560) | 135 | T1W+C | 5.3/2.2 | 340×380 | 512×512 | 2 | 2 | 60 | 8° | 4min16s | 6 |
|  |  | T2W | 4000/83.2 | 340×320 | 364×364 | 5 | 1.5 | 35 | 90° | 2min9s | 1 |
|  |  | DWI | 9965/101.5 | 320×320 | 240×240 | 6 | 1.5 | 36 | 90° | 1min36s | 1 |
| Philip 3.0T  (Ingenia) | 93 | T1W+C | 4.5/1.5 | 320×320 | 384×384 | 1.5 | 1 | 48 | 10° | 4min15s | 9 |
|  |  | T2W | 6214/75.2 | 380×340 | 380×380 | 7 | 1 | 24 | 90° | 2min1s | 1 |
|  |  | DWI | 4965/75 | 320×320 | 200×200 | 7 | 1 | 24 | 90° | 1min25s | 1 |

**Table S2.** Performance for different classifiers on pre-ablation MR sequences in primary cohorts

| **Classifiers** | **MRI Sequence** | **AUC (95% CI)** |
| --- | --- | --- |
| LR | T1WI+C | 0.790 (0.725-0.855) |
|  | T2WI | 0.768 (0.731-0.805) |
|  | DWI | 0.768 (0.738-0.798) |
| LDA | T1WI+C | 0.799 (0.750-0.850) |
|  | T2WI | 0.771 (0.732-0.810) |
|  | DWI | 0.759 (0.719-0.800) |
| GBDT | T1W+C | 0.800 (0.762-0.838) |
|  | T2WI | 0.790 (0.761-0.819) |
|  | DWI | 0.773 (0.716-0.830) |
| SVM | T1WI+C | **0.801 (0.756-0.846)** |
|  | T2WI | **0.793 (0.725-0.861)** |
|  | DWI | **0.790 (0.735-0.845)** |
